# A novel antidepressant mechanism of baicalin: enhancing KIF5A-mediated axoplasmic transport and vesicular trafficking in glutamatergic neurons

**DOI:** 10.3389/fphar.2025.1577676

**Published:** 2025-04-01

**Authors:** Shuaifei Lu, Jiduo Shen, Xiaohui Jin, Changjing Zhang, Baoying Wang, Xianghua Liu, Ming Bai, Erping Xu, Xiangli Yan, Yucheng Li

**Affiliations:** ^1^ Collaborative Innovation Center of Research and Development on the Whole Industry Chain for Yu-Yao of Henan Province, Henan University of Chinese Medicine, Zhengzhou, Henan, China; ^2^ Academy of Chinese Medical Sciences, Henan University of Chinese Medicine, Zhengzhou, Henan, China; ^3^ College of Pharmacy, Henan University of Chinese Medicine, Zhengzhou, Henan, China

**Keywords:** depression, baicalin, axoplasmic transport, synaptic vesicles, KIF5A

## Abstract

**Introduction:**

Dysfunction of axoplasmic transport is closely linked to depression. Baicalin, a major flavonoid in *Scutellaria baicalensis*, a well-known traditional Chinese medicine used in depression treatment, has demonstrated antidepressant-like effects in previous studies. However, its potential role in regulating axoplasmic transport has not been explored. This study aims to investigate the antidepressant mechanisms of baicalin through modulation of axoplasmic transport in hippocampal neurons.

**Methods:**

Male C57BL/6N mice were exposed to chronic unpredictable mild stress (CUMS) and treated with baicalin (10, 20, 40 mg/kg) or fluoxetine (20 mg/kg). Depression-like behaviors were assessed using the sucrose preference test (SPT), forced swimming test (FST), tail suspension test (TST), and locomotor activity test (LAT). Hippocampal neuronal pathology was examined using transmission electron microscopy (TEM), Nissl, and Golgi staining. Transcriptomic analysis was conducted to explore the molecular mechanisms of baicalin. HT22 cells were cultured *in vitro* and treated with corticosterone (CORT) and baicalin. FM1-43 was used to label vesicles and track vesicular movement. mRNA and protein levels were measured by qRT-PCR, Western blotting, and immunofluorescence.

**Results:**

Baicalin significantly alleviated CUMS-induced depressive behaviors, increasing sucrose preference, reducing immobility time in TST and FST, and increasing food intake without affecting locomotor activity. It improved hippocampal CA3 neuronal damage, increased dendritic spine density, and promoted presynaptic vesicle accumulation, particularly in glutamatergic neurons. Transcriptomic analysis revealed that baicalin upregulated vGLUT2 (encoded by the Slc17a6 gene) and significantly increased the expression of GluN2B, GluA1, and PSD95. Moreover, baicalin upregulated the expression of kinesin family member 5A (KIF5A) both *in vivo* and *in vitro*, enhancing vesicle movement along axons and increasing vesicle-associated membrane protein 2 (VAMP2) enrichment in synaptosomes.

**Discussion:**

These findings suggest that baicalin enhances anterograde axoplasmic transport by upregulating KIF5A expression, facilitating vesicular trafficking and improving synaptic function in glutamatergic neurons. This study provides novel insights into the molecular mechanisms of antidepressant effects of baicalin, highlighting KIF5A as a potential therapeutic target for depression.

## 1 Introduction

Depression is a prevalent mental illness that severely impacts individuals’ quality of life. It is estimated that over 300 million people suffer from depression globally, and it has become a leading cause of global health burden, contributing to direct economic losses of up to $1 trillion annually, which highlights the urgent need for effective interventions (Cai et al., 2023; Marx et al., 2023). Although extensive research has been conducted to understand the underlying mechanisms of depression, its precise pathogenesis remains unclear, and the development of novel antidepressant treatments has been progressing slowly (Marwaha et al., 2023; Steffens, 2024).

A growing body of evidence demonstrates that depression is fundamentally associated with synaptic dysfunction (Duman and Aghajanian, 2012; Rantamäki and Kohtala, 2020). Axoplasmic transport, the intracellular process responsible for the directed movement of cellular components along axons, is critical for maintaining synaptic function (Guedes-Dias and Holzbaur, 2019). This transport mechanism involves ATP-dependent anterograde and retrograde transport of various cargoes, including mitochondria, neurotransmitters, and proteins, ensures proper neuronal communication by delivering these materials to the axon terminal or soma (Maday et al., 2014). Both clinical and animal studies have demonstrated the disruptions in this progress have been implicated in various neuropsychiatric disorders, including depression and Alzheimer’s disease (Badal and Puthanveettil, 2022; Bakhtiarzadeh et al., 2018; Berth and Lloyd, 2023). Enhancing this transport has been shown to improve neuronal communication and alleviate depressive symptoms (Badal and Puthanveettil, 2022; Shah and Goldberg, 2018).

Kinesin is a crucial superfamily of anterograde motor proteins responsible for transporting cellular components from the neuronal soma to the axon terminal (Guillaud et al., 2020; Hirokawa et al., 2010). Kinesin-1, previously known as conventional kinesin, is a heterotetramer composed of two kinesin heavy chains (KIF5A, KIF5B, or KIF5C) and two kinesin light chains (Hirokawa and Noda, 2008). KIF5A plays a key role in the transport of mitochondria, vesicles, and proteins essential for synaptic function, and defects in its expression or function can lead to impaired neuronal communication (Karle et al., 2012). Recent study has shown KIF5A mediates AMPA receptor synaptic removal during long-term depression (Brachet et al., 2021), suggesting a potential link between disrupted KIF5A-mediated axoplasmic transport and depression.

Baicalin, a major flavonoid compound derived from *Scutellaria baicalensis*, has demonstrated antidepressant-like effects in various animal models of depression (Guo et al., 2019; Li et al., 2013; Yi et al., 2024). Recent studies suggest that baicalin can enhance the vesicular transport and release of dopamine (DA), indicating its potential role in improving axoplasmic transport (Zhou et al., 2019). However, whether baicalin exerts its antidepressant effects through modulation of axoplasmic transport remains unexplored. Therefore, the present study aims to investigate the effects of baicalin on axoplasmic transport in hippocampal neurons and to explore its underlying antidepressant mechanisms.

## 2 Materials and methods

### 2.1 Chemicals and reagents

Baicalin (98% purity) was purchased from Aladdin Co., Ltd. (Shanghai, China). Fluoxetine hydrochloride was purchased from Lilly Co., Ltd. (Suzhou, Jiangsu Province, China). Corticosterone (CORT, ≥98% purity) and dimethyl sulfoxide (DMSO) were purchased from Sigma-Aldrich (Saint Louis, MO, United States). The BCA protein assay kit and RIPA lysis buffer were obtained from CWBIO (Beijing, China). Primary rabbit anti-GAPDH (KANGCHEN, Shanghai, China), rabbit anti-KIF5A and VAMP2 (ABclonal, Wuhan, China), mouse anti-Dynein (Sigma-Aldrich, Saint Louis, MO, United States); Secondary antibodies including goat anti-rabbit IgG (KANGCHEN, Shanghai, China), goat anti-mouse IgG (ABclonal, Wuhan, China), goat anti-rabbit IgG Alexa Fluor 488 (Thermo Fisher, Waltham, MA, United States). Fetal bovine serum (FBS) was purchased from Thermo Fisher (Waltham, MA, United States). FM1-43 was obtained from Biotium (Fremont, CA, United States). The CCK-8 kit was purchased from Dojindo (Kumamoto, Japan). DMEM, penicillin-streptomycin solution, and trypsin were provided by Cytiva (Marlborough, MA, United States).

### 2.2 Animals

Male C57BL/6N mice (16–20 g) were purchased from Beijing Vital River Laboratory Animal Technology (Beijing, China). The mice were housed under a 12 h light/dark cycle at 23°C–25°C and 40%–60% humidity. Prior to the experiment, mice were acclimatized for 1 week with *ad libitum* access to food and water. All animal procedures were performed following the National Institutes of Health guide for the care and use of Laboratory animals (NIH Publications No. 8023, revised 1978), compliant with the ARRIVE (Animal Research: Reporting of *In Vivo* Experiments) guidelines, and were approved by the Committee of Animal Care of Henan University of Chinese Medicine (DWLLGZR202202033).

### 2.3 Experimental design

**FIGURE 1:**
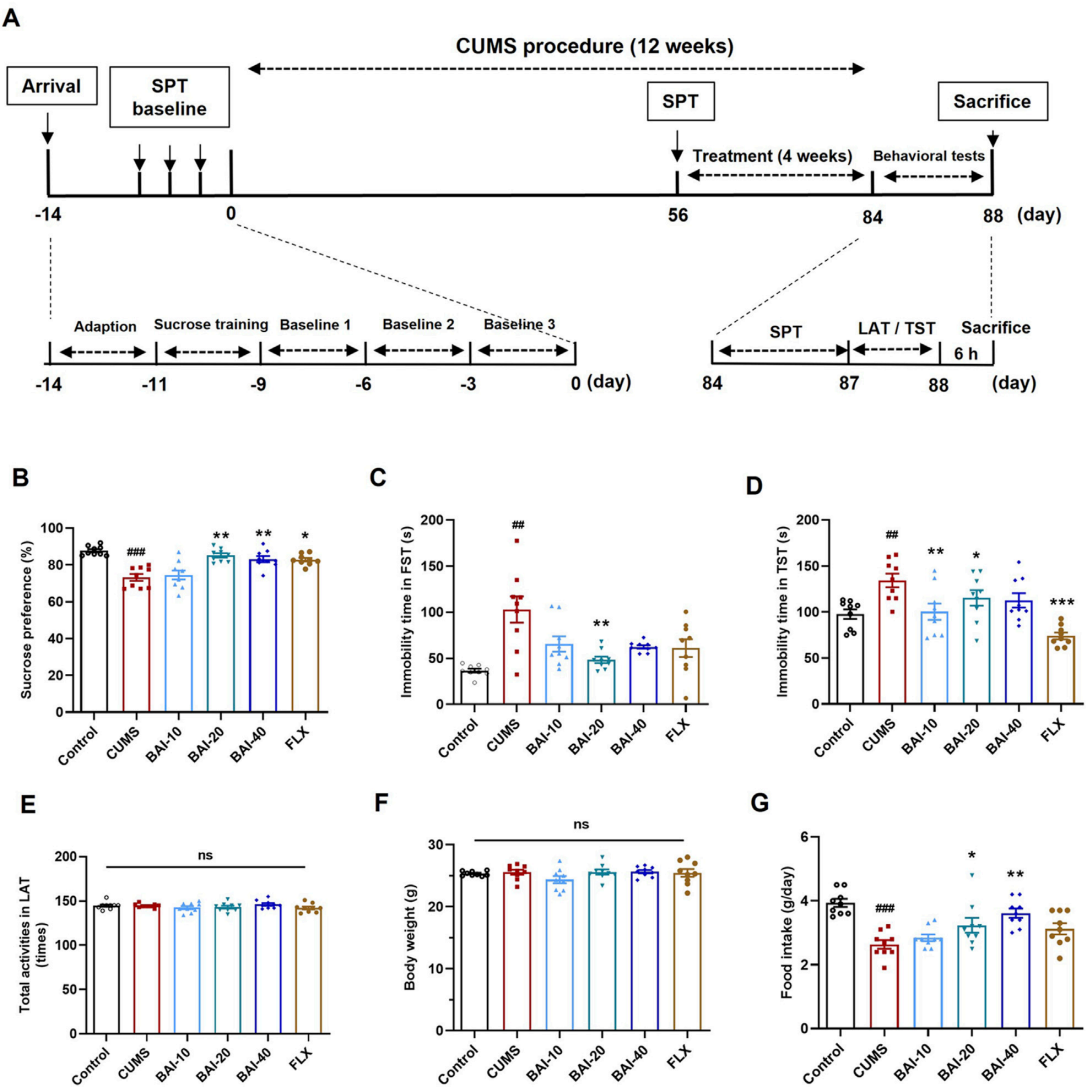
Baicalin reverses depression-like behaviors induced by CUMS in mice. **(A)** The schedule of animal experiments. **(B)** Sucrose preference. **(C)** Immobility time in the FST. **(D)** Immobility time in the TST. **(E)** Total number of locomotor activity in the LAT. **(F)** Body weight. **(G)** Daily food intake. Data are presented as means ± SEM and analyzed by one-way ANOVA with Tukey’s *post hoc* test. n = 9. ^#^ *p* < 0.05, ^##^ *p* < 0.01, and ^###^ *p* < 0.001 compared with control group; *p < 0.05, **p < 0.01, and ***p < 0.001 compared with CUMS group. ns: no statistical significance. BAI-10: 10 mg/kg baicalin; BAI-20: 20 mg/kg baicalin; BAI-40: 40 mg/kg baicalin; FLX: 20 mg/kg fluoxetine.

After a week of acclimatization, the sucrose preference test (SPT) was conducted three times to exclude mice that were insensitive to sucrose (sucrose preference <60%). The remaining mice were randomly assigned to two groups: control (9 mice) and stress (45 mice) groups. The stress group underwent the chronic unpredictable mild stress (CUMS) procedure for 8 weeks, while the control group was housed in a separate room without any stressors. At the end of week 8, a final sucrose preference test was performed to assess depression-like behaviours, and the stress group was subdivided into 5 treatment groups based on equal sucrose preference (9 mice per group): CUMS, BAI-10 (10 mg/kg baicalin), BAI-20 (20 mg/kg baicalin), BAI-40 (40 mg/kg baicalin), and FLX (20 mg/kg fluoxetine). All drugs were suspended in 5% CMC-Na solution and administered by oral gavage at 10 mL/kg for 4 weeks. The CUMS and control groups received an equal volume of 5% CMC-Na solution. The dose of baicalin was selected based on our previous study (Li et al., 2013; Li et al., 2015; Liu et al., 2024). The CUMS procedure continued throughout the drug administration period. A schematic of the experimental timeline is shown in Figure 1A.

### 2.4 CUMS procedure

The CUMS procedure was performed following the method described in our previous study (Jin et al., 2023). Briefly, mice were individually housed in cages (310 × 230 × 157 mm) and exposed to 1–2 different stressors each day, including the reversal of light/dark cycle (24 h), food deprivation (12 h), water deprivation (12 h), tilting of the cage (45° for 24 h), cold (4°C) or hot (45°C) water swimming (4 min), white noise (90 dB for 12 h), soiled cage (24 h), restraint (3 h), flashlight exposure (150 flashes/min for 6 h), and pair-housing (2 h). All stressors were applied unpredictably and randomly to ensure variability in stress exposure.

### 2.5 Behavioral tests

#### 2.5.1 Sucrose preference test

The formal SPT was conducted at the end of weeks 8 and 12, following a protocol modified from Liu et al. (2018). Briefly, mice were first trained to consume a 1% sucrose solution for 48 h. After training, mice were deprived of food and water for 12 h, followed by the placement of one bottle of sucrose solution and one bottle of fresh water for 12 h. The positions of the bottles were switched halfway through the test to eliminate any positional preference. After 12 h, the bottles were weighed, and sucrose preference was calculated as:

$$\text{sucrose preference }\left(\%\right)=\left[\text{sucrose intake }/\;\left(\text{sucrose intake}+\text{water intake}\right)\right]\times 100\%.$$

#### 2.5.2 Tail suspension test

Mice were suspended by the tail 1 cm from the tip, and their immobility was recorded over 6 min using a video camera. Immobility time was measured during the final 4 min of the test, where immobility was defined as passive hanging without movement.

#### 2.5.3 Forced swimming test

Mice were placed in a cylindrical tank filled with water (25°C) to a depth of 15 cm, where they were allowed to swim freely for 6 min, and the immobility time was measured during the final 4 min of the test.

#### 2.5.4 Locomotor activity test

Locomotor activity was assessed using an infrared sensor-based locomotor activity tester (TECHMAN, Chengdu, China). An infrared camera positioned 30 cm above the cage detected the total movement of the mice over a 5-min period in a dark environment.

### 2.6 Tissue sample collection

Twenty-four hours after the behavioural tests, all mice were sacrificed by decapitation following anaesthesia with tribromoethanol (400 mg/kg, i. p.). The brain tissues were rapidly dissected, and six mice from each group were randomly selected. Their whole brains were fixed in 4% paraformaldehyde, with three brains used for Nissl staining and the other three for Golgi staining. The remaining three mice were used for hippocampal tissue dissection for transmission electron microscopy (TEM) observation.

Since the hippocampal samples from this batch of mice were insufficient to assess all parameters, a second batch of animal models was prepared using the same method. The groups were as follows: Control, CUMS, BAI-20, and FLX-20, with 12 mice per group. In the second batch, three mice per group were used for transcriptomic analysis, three for immunoelectron microscopy, and the remaining six mice were divided into left and right hippocampal dissection, with left for qPCR and right for Western blot analysis. The behavioural evaluation results of the second batch of animals are shown in Supplementary Figure 1.

### 2.7 Nissl staining

The whole brains were dissected and fixed in 4% paraformaldehyde for 24 h. After fixation, the brains were embedded in paraffin and dehydrated. Thin sections (5 μm) were cut and stained with Nissl staining solution. The slides were then scanned using a Digital Pathology Slide Scanner (KF-PRO-005-EX, KFBIO, Hangzhou, China).

### 2.8 Golgi staining

The brains were immersed in Golgi stain for 14 days, with the dye replaced every 2 days. After three washes, the brain was incubated in glacial acetic acid overnight, then transferred to a 30% sucrose solution for cryoprotection. Thin slices (100 μm) were sectioned using a vibratome. Dendritic structures were observed under a microscope. For analysis, nine dendrites from the hippocampal CA3 region of each slide were randomly selected, and the number of spines per 10 μm was quantified.

### 2.9 Transmission electron microscopy

Hippocampal tissues were rapidly dissected, cut into 1 mm^3^ pieces, and fixed overnight in 4% glutaraldehyde at 4°C. After post-fixation with 1% osmic acid and dehydration, the samples were embedded in epoxy resin. Ultrathin sections were stained with uranyl acetate and lead citrate, and the ultrastructure of neuronal synapses was observed under a transmission electron microscope (JEM-1400, JEOL, Akishima, Japan).

### 2.10 Immunoelectron microscopy

Hippocampal tissues were quickly dissected, fixed in 4% paraformaldehyde for 1 h, and stored overnight in 2% paraformaldehyde at 4°C. After washing, tissues were permeabilized with PBS containing 5% BSA, 3% goat serum, and 0.04% Triton-X 100. They were incubated overnight with anti-VGLUT2 antibody (1:2,000) at 4°C, followed by biotinylated secondary antibody. Immunolabeling was detected using avidin-biotin complex markers. After DAB staining, tissues were post-fixed with osmic acid, stained with uranyl acetate, and dehydrated in graded ethanol. The samples were embedded in epoxy resin, sectioned, and observed under a transmission electron microscope for immunolabeling.

### 2.11 Transcriptomic analysis

Nine mice from the Control, CUMS, and BAI-20 groups (3 mice per group) were randomly selected for hippocampal tissue RNA sequencing, performed by Hangzhou Lianchuan Biotechnology Co., Ltd. Total RNA was extracted using the Trizol method, and mRNA was enriched with oligo(dT)-attached magnetic beads. The mRNA was fragmented and reverse transcribed into cDNA, followed by double-strand DNA synthesis using *E. coli* DNA polymerase I and RNase H. The double-stranded ends were converted to blunt ends with dUTPs, and adapters were added. The fragments were purified and PCR amplified to generate a library with a size of 300 ± 50 bp, then sequenced using the Illumina Novaseq™ 6000 system.

Differentially expressed genes (DEGs) were selected based on a fold change (FC) > 2 or FC < 0.5 and a p-value < 0.05. Gene names were converted to UniProt IDs using UniProt (https://www.uniprot.org/). GO annotation and KEGG pathway enrichment were performed using the DAVID database (https://david.ncifcrf.gov/home.jsp). Data visualization was conducted using the OmicStudio platform (https://www.omicstudio.cn/tool). The raw transcriptomic sequencing data have been deposited in the NCBI Sequence Read Archive (SRA) public database (PRJNA1219842).

### 2.12 Quantitative real-time polymerase chain reaction

Total RNA was extracted from hippocampus using an RNA extraction kit (Tiangen, Beijing, China), and cDNA was synthesized using the FastKing RT kit (Tiangen, Beijing, China). qPCR was performed on an ABI-Q6 system (Applied Biosystems, Waltham, MA, United States) using a SYBR Green kit (Qiagen, Hilden, Germany). GAPDH was used as internal reference gene, and data were analyzed using the 2^−ΔΔCT^ method (Livak and Schmittgen, 2001). The primers were synthesized by Invitrogen (Shanghai, China), and the sequences are listed in Table 1.

**TABLE 1:** The sequences of primers for qPCR.

Gene	Accession no.	Primers (5′–3′)	Product (bp)
*Gapdh*	NM_001289726.1	Forward: TCTCCTGCGACTTCAACA	117
		Reverse: TGT​AGC​CGT​ATT​CAT​TGT​CA	
*Kif5a*	NM_008447.4	Forward: TCC​TGC​TTG​GCC​CTG​TAT​G	145
		Reverse: GGT​CAG​GGT​TGG​CTA​TGA​ATG	
*Kif5b*	NM_008448.3	Forward: CTT​ACC​CCA​AGA​CTG​AGC​GG	92
		Reverse: CTT​TGA​TGT​TGC​ACT​CCG​CC	
*Kif5c*	NM_008449.3	Forward: AAG​TCT​CTC​GTG​AAC​CGC​AG	127
		Reverse: TGA​TCT​TGG​CTT​CGT​GCT​GT	
*Dync1i1*	NM_001191023.1	Forward: CCC​GTT​CAA​GAT​GAC​TCC​GA	102
		Reverse: CAA​CGG​CTG​CAC​TAG​AGG​G	
*Myo5a*	NM_010864.2	Forward: GAA​TGA​GTT​GCG​CAA​AGC​CC	191
		Reverse: TGT​CAT​CCT​TGG​GTT​GGA​TGG	
*Mapt*	NM_001285455.1	Forward: CCT​CTT​CTG​TCC​TCG​CCT​TC	155
		Reverse: CCA​CAC​GAG​CTT​TTA​AGC​CA	

### 2.13 Isolation of hippocampal synaptosomes

According to a previous method (Speed et al., 2015), synaptosomes were isolated from mouse hippocampus using Syn-PER reagent (Thermo, Waltham, MA, United States). Briefly, hippocampal tissue was homogenized in Syn-PER reagent with protease/phosphatase inhibitor. After centrifugation at 1,200 × g for 10 min at 4°C, the supernatant was centrifuged at 15,000 × g for 20 min again. The precipitate (synaptosomal fraction) was collected and lysed in RIPA buffer for subsequent Western blot analysis. Synaptosomes were validated by the enrichment of synaptophysin and postsynaptic density 95 (PSD95) markers.

### 2.14 Western blot

Hippocampal tissues were homogenized in RIPA lysate containing cOmplete™ ULTRA protease inhibitors (Roche, Shanghai, China) and PhosSTop™ phosphatase inhibitor cocktail (Roche, Shanghai, China). The homogenate was centrifuged at 12,000 × g for 20 min and supernatants were collected. The protein concentration was determined using the BCA protein assay kit. The protein samples (10 μg) were separated by sodium dodecyl sulfate-polyacrylamide gel electrophoresis (SDS‒PAGE) and transferred onto polyvinylidene difluoride (PVDF) membranes (Millipore, Shanghai, China). After blocking with 5% non-fat milk, the membranes were incubated with primary antibodies against GAPDH (1:60,000), KIF5A (1:1,000), and VAMP2 (1:6,000) overnight at 4°C. After washing, membranes were incubated with secondary antibodies for 30 min at room temperature and visualized using enhanced chemiluminescence (ECL) reagent (Merck, Darmstadt, Germany). GAPDH was used to normalize total proteins and PSD95 was used for synaptosome-enriched protein normalization.

### 2.15 Cell culture and CCK-8 activity

HT22 mouse hippocampal neurons were cultured in DMEM supplemented with 10% FBS and 1% penicillin/streptomycin at 37°C in a 5% CO_2_ incubator. For cell viability assessment, HT22 cells (1 × 10^4^ cells/well) were seeded in 96-well plates and cultured for 24 h, and the culture medium was replaced with a drug-containing medium devoid of FBS for an additional 24 h. Subsequently, the medium was replaced with serum-free medium containing 10% CCK-8 reagent for 1 h. Absorbance was measured at 450 nm using a microplate reader.

### 2.16 FM1-43 staining

HT22 cells (4 × 10^4^ cells/dish) were seeded in 20 mm dishes and cultured in DMEM supplemented with 10% FBS for 24 h, and the medium was replaced with a drug-containing medium for an additional 24 h. The cells were then washed twice with Hank’s Balanced Salt Solution (HBSS) and stained with 4 μM FM1-43 in HBSS for 2 min. After staining, cells were washed twice with HBSS to remove excess dye. Fluorescent imaging was performed using an inverted fluorescence microscope (TS-2, Nikon, Japan) under a 455 nm excitation filter for 1 min to capture continuous dynamic images of FM1-43 fluorescence.

### 2.17 Immunofluorescence

HT22 cells were seeded on glass coverslips and cultured for 24 h before the medium was replaced with drug-containing media for another 24 h. Following treatment, the cells were washed twice with Dulbecco’s phosphate-buffered saline (DPBS) and fixed in 4% paraformaldehyde for 15 min. Blocking was performed with 3% BSA dissolved in DPBS at room temperature for 30 min. Primary antibodies against KIF5A (1:100) and VAMP2 (1:100) were diluted in DPBS and incubated with the coverslips overnight at 4°C. After washing three times, the coverslips were incubated with goat anti-rabbit IgG Alexa Fluor 488 secondary antibody (1:500) for 1 h at room temperature, and then stained with Hoechst 33,342 (Beyotime, Shanghai, China) for 20 min to label cell nuclei. After washing, the coverslips were mounted with anti-fade mounting medium and imaged using a confocal laser scanning microscope (LSM780, Zeiss, Germany).

### 2.18 Statistical analysis

Data were presented as the mean ± SEM and analyzed using SPSS 24.0 software (IBM Corp, Armonk, NY, United States). One-way ANOVA followed by Tukey’s *post hoc* test was used for multiple comparisons. Normality of data distribution was assessed using the Shapiro-Wilk test, confirming that the data followed a normal distribution. Statistical significance was set at *p* < 0.05.

## 3 Results

### 3.1 Baicalin reverses depression-like behaviours induced by CUMS

To evaluate the effects of baicalin on depression-like behaviours induced by CUMS, a series of behavioural tests were conducted, including the SPT, FST, TST, and the LAT. CUMS exposure significantly reduced sucrose preference and increased the immobility times in both the FST and TST, which were effectively reversed by baicalin treatment, particularly at a dose of 20 mg/kg (Figures 1B–D). Specifically, fluoxetine only reversed behavioural changes in the SPT and TST, without significantly improving the results in the FST. Furthermore, baicalin did not affect spontaneous locomotor activity or body weight (Figures 1E, F). Additionally, both 20 mg/kg and 40 mg/kg doses of baicalin restored the reduced food intake induced by CUMS, whereas fluoxetine did not exhibit a similar effect (Figure 1G).

### 3.2 Baicalin ameliorates pathological changes in hippocampal neurons induced by CUMS and promotes synaptic vesicle accumulation

**FIGURE 2:**
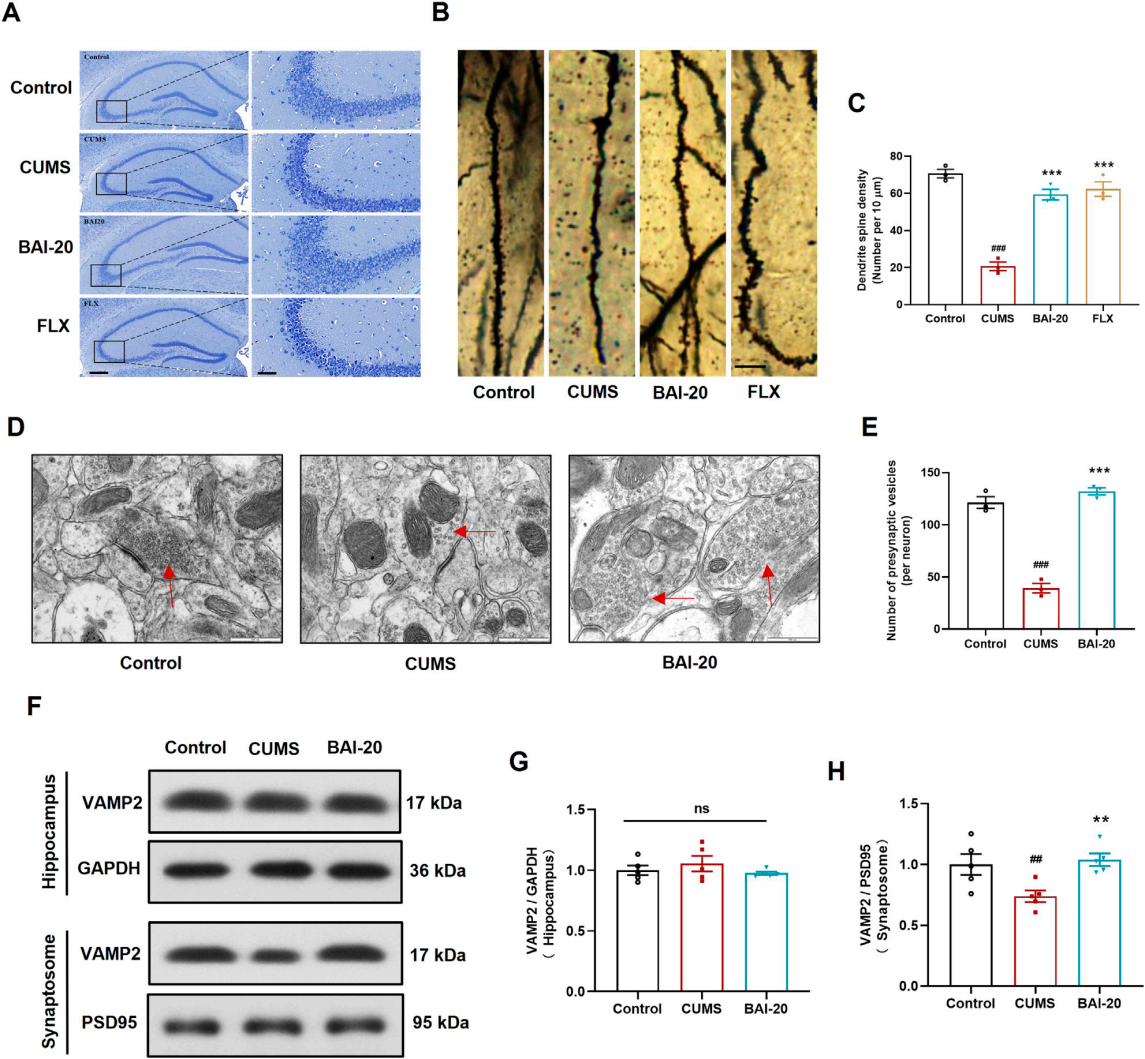
Baicalin ameliorates hippocampal neuronal pathology induced by CUMS. **(A)** Nissl staining. Scale bar: 200 μm (left, ×50) and 50 μm (right, ×200). **(B)** Golgi staining. Scale bar: 10 μm. **(C)** Quantification of dendritic spines per 10 μm (10 dendrites were randomly selected per slide). **(D)** Synapse in the hippocampal CA3 region observed by transmission electron microscopy (×50,000). Red arrows indicate presynaptic vesicles. Scale bar: 500 nm. **(E)** Quantification of presynaptic vesicles (10 complete synapses per slide were randomly selected for counting). **(F)** Immunoblot analysis of VAMP2 expression in the hippocampus and synaptosome. GAPDH was used as a reference control for hippocampal tissue, and PSD95 for synaptosomes. **(G)** Relative quantification of VAMP2 proteins in the hippocampus (n = 5). **(H)** Relative quantification of VAMP2 proteins in synaptosomes (n = 5). Data are presented as means ± SEM and analyzed by one-way ANOVA with Tukey’s *post hoc* test. ^#^ *p* < 0.05, ^##^ *p* < 0.01, and ^###^ *p* < 0.001 compared with control group; *p < 0.05, **p < 0.01, and ***p < 0.001 compared with CUMS group. ns: no statistical significance. BAI-20: 20 mg/kg baicalin; FLX: 20 mg/kg fluoxetine.

To investigate the effects of baicalin on neuronal pathology, Nissl staining, Golgi staining, and TEM were performed to examine the morphology of neurons in the CA3 region of the hippocampus. The results demonstrated that baicalin significantly alleviated neuronal shrinkage in the CA3 region of the hippocampus induced by CUMS and enhanced dendritic spine density (Figures 2A–C). Additionally, baicalin treatment notably increased the number of presynaptic vesicles (Figures 2D, E). To further validate this effect, we assessed the protein expression of VAMP2, a well-established marker of synaptic vesicles. Baicalin did not alter the expression of VAMP2 in total hippocampal proteins, but significantly increased VAMP2 levels in synaptosomes (Figures 2F–H), suggesting that baicalin promotes the accumulation of synaptic vesicles at presynaptic terminals. The synaptosome protein markers PSD95 were predominantly enriched in the isolated synaptosomes, confirming the successful isolation and availability of the synaptosomes for analysis (Supplementary Figure 2).

### 3.3 Baicalin reverses the decrease in hippocampal Slc17a6 expression induced by CUMS

**FIGURE 3:**
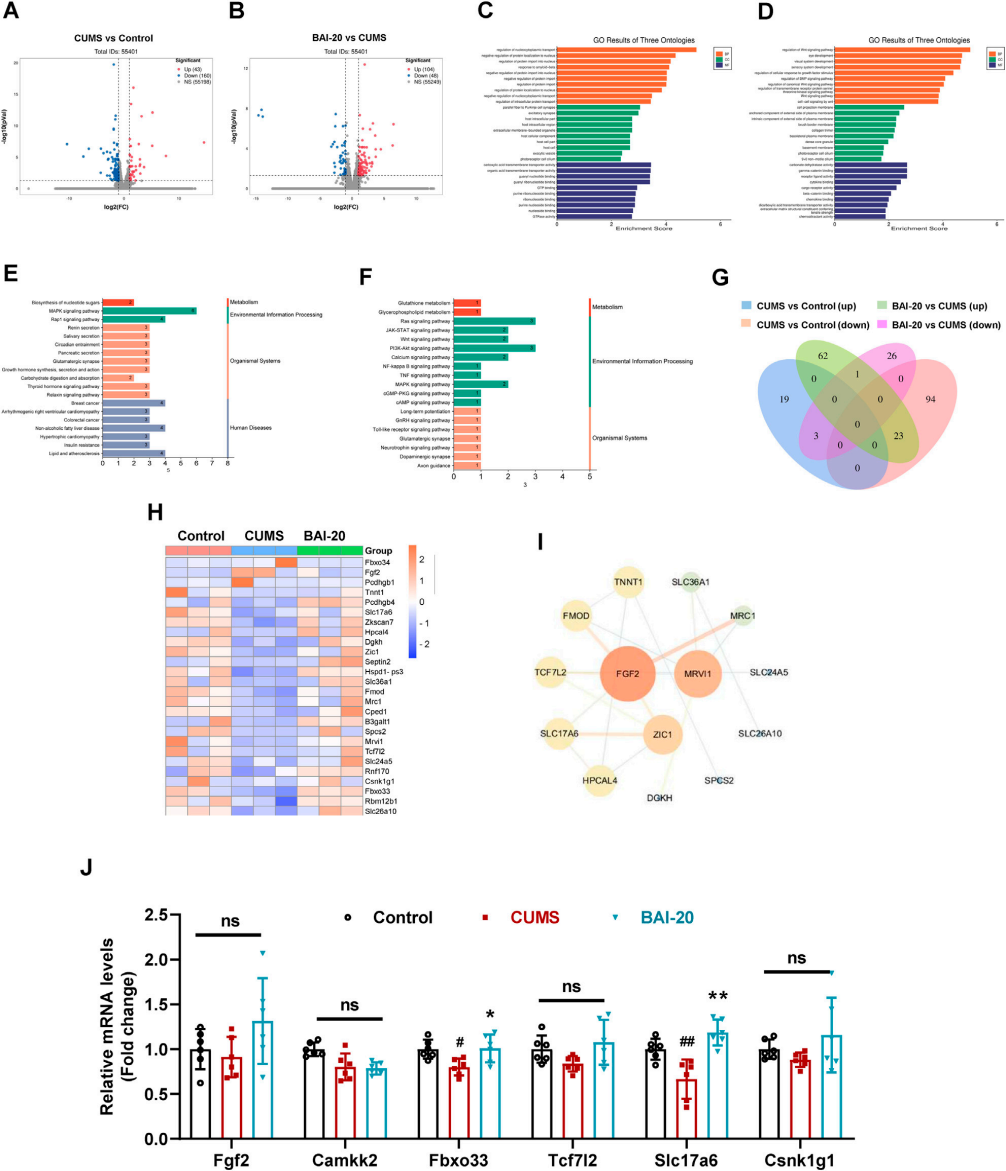
Effects of Baicalin on hippocampal transcriptomic changes. **(A)** Volcano plot of DEGs between the control and CUMS groups. **(B)** Volcano plot of DEGs between the CUMS and BAI-20 groups. **(C)** GO analysis of DEGs between the control and CUMS groups. **(D)** GO analysis of DEGs between the CUMS and BAI-20 groups. **(E)** KEGG analysis of DEGs between the control and CUMS groups. **(F)** KEGG analysis of DEGs between the CUMS and BAI-20 groups. **(G)** Venn diagram. **(H)** Hierarchical clustering analysis. **(I)** PPI network analysis. **(J)** qPCR validation of DEGs (n = 6). Data are presented as means ± SEM and analyzed by one-way ANOVA with Tukey’s *post hoc* test. ^#^ *p* < 0.05 and ^##^ *p* < 0.01 compared with control group; *p < 0.05 and **p < 0.01compared with CUMS group. ns: no statistical significance. BAI-20: 20 mg/kg baicalin.

To investigate the mechanism by which baicalin promotes synaptic vesicle accumulation, we conducted transcriptomic analysis to reveal the changes in hippocampal mRNA expression. The results revealed that CUMS induced the upregulation of 43 genes and downregulation of 160 genes, while baicalin treatment led to the upregulation of 104 genes and downregulation of 48 genes compared to the CUMS group (Figures 3A, B). GO enrichment analysis indicated that the DEGs were predominantly involved in processes such as nucleocytoplasmic transport, intracellular protein transport, excitatory synapse function, and exocytic vesicle trafficking (Figures 3C, D). KEGG pathway analysis revealed that both CUMS and baicalin treatment influenced glutamatergic synapses (Figures 3E, F). Venn analysis of the DEGs identified 26 genes exhibiting opposite expression trends between the two groups (Figure 3G). Cluster analysis (Figure 3H) and PPI analysis (Figure 3I) highlighted key candidate genes for further validation. qPCR validation showed that Slc17a6, which encodes vGLUT2, a presynaptic marker for glutamate vesicles, was significantly downregulated in the CUMS group but markedly upregulated in the baicalin-treated group (Figure 3J).

### 3.4 Baicalin increases the accumulation of synaptic vesicles in hippocampal glutamatergic neurons

**FIGURE 4:**
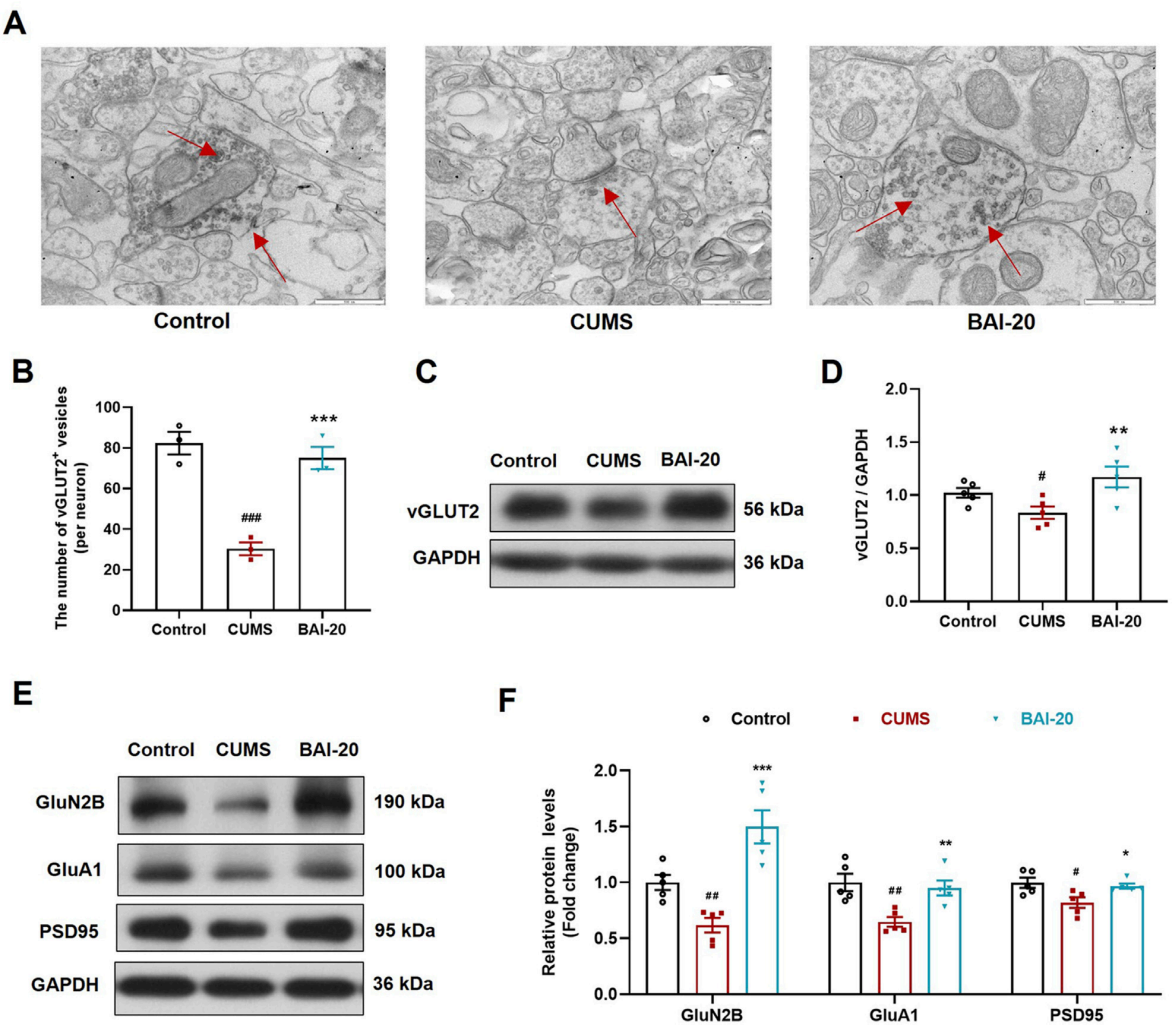
Baicalin increases the accumulation of synaptic vesicles in glutamatergic neurons. **(A)** Immunoelectron microscopy of glutamatergic synapses (red arrows indicate vGLUT2+ glutamatergic neurons, ×50,000). Scale bar: 500 nm. **(B)** Quantification of vGLUT2+ vesicles (10 complete synapses per slide were randomly selected for counting). **(C)** Immunoblot analysis of vGLUT2 in the hippocampus. **(D)** Quantification of VAMP2 protein in the hippocampus (n = 5). **(E)** Immunoblot analysis of GluN2B, GluA1, and PSD95 in the hippocampus. **(F)** Quantification of GluN2B, GluA1, and PSD95 proteins in the hippocampus (n = 5). Data are presented as means ± SEM and analyzed by one-way ANOVA with Tukey’s *post hoc* test. ^#^ *p* < 0.05, ^##^ *p* < 0.01, and ^###^ *p* < 0.001 compared with control group; *p < 0.05, **p < 0.01, and ***p < 0.001 compared with CUMS group. BAI-20: baicalin (20 mg/kg).

To verify whether the observed synaptic vesicle accumulation in TEM corresponded to glutamatergic synapses, we performed immunoelectron microscopy to label vGLUT2. The results showed that vGLUT2-positive vesicles were significantly reduced in the CUMS group, whereas baicalin treatment led to a notable increase in these vesicles (Figures 4A, B), consistent with the TEM observations. Furthermore, protein expression analysis of vGLUT2 confirmed this result (Figures 4C, D). The expression of GluN2B, GluA1, and PSD95 was significantly reduced In the CUMS group, while baicalin treatment significantly increased their levels (Figures 4E, F), suggesting that baicalin enhances synaptic transmission and potentiates postsynaptic responses. These findings imply that the observed vesicle accumulation is not due to inhibitory release, but rather an enhancement of synaptic vesicle activity.

### 3.5 Baicalin increases the expression of KIF5A and enhances axoplasmic transport in the hippocampus

**FIGURE 5:**
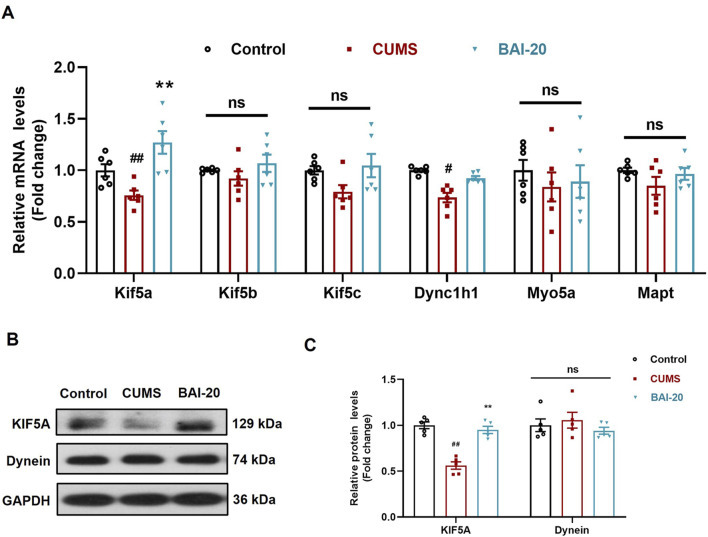
Baicalin upregulates the mRNA and protein expression of KIF5A in the hippocampus. **(A)** qPCR analysis (n = 6). **(B)** Immunoblot analysis of KIF5A and Dynein proteins in the hippocampus. **(C)** Quantification of KIF5A and Dynein proteins (n = 5). Data are presented as means ± SEM and analyzed by one-way ANOVA with Tukey’s *post hoc* test. ^#^ *p* < 0.05 and ^##^ *p* < 0.01 compared with control group; *p < 0.05 and **p < 0.01compared with CUMS group. ns: no statistical significance. BAI-20: baicalin (20 mg/kg).

To investigate the effect of baicalin on axoplasmic transport, we detected the mRNA and protein levels of key axonal transport genes in the hippocampus. The results revealed that the expression of KIF5A, a critical motor protein involved in anterograde axoplasmic transport, was significantly reduced in the CUMS group, but was markedly upregulated in the baicalin-treated group (Figures 5A–C). These findings suggest that baicalin may enhance anterograde axoplasmic transport by upregulating KIF5A expression, thereby facilitating the transport of vesicles to the synaptic terminals.

### 3.6 Baicalin enhances KIF5A-mediated vesicular transport in HT22 cells

**FIGURE 6:**
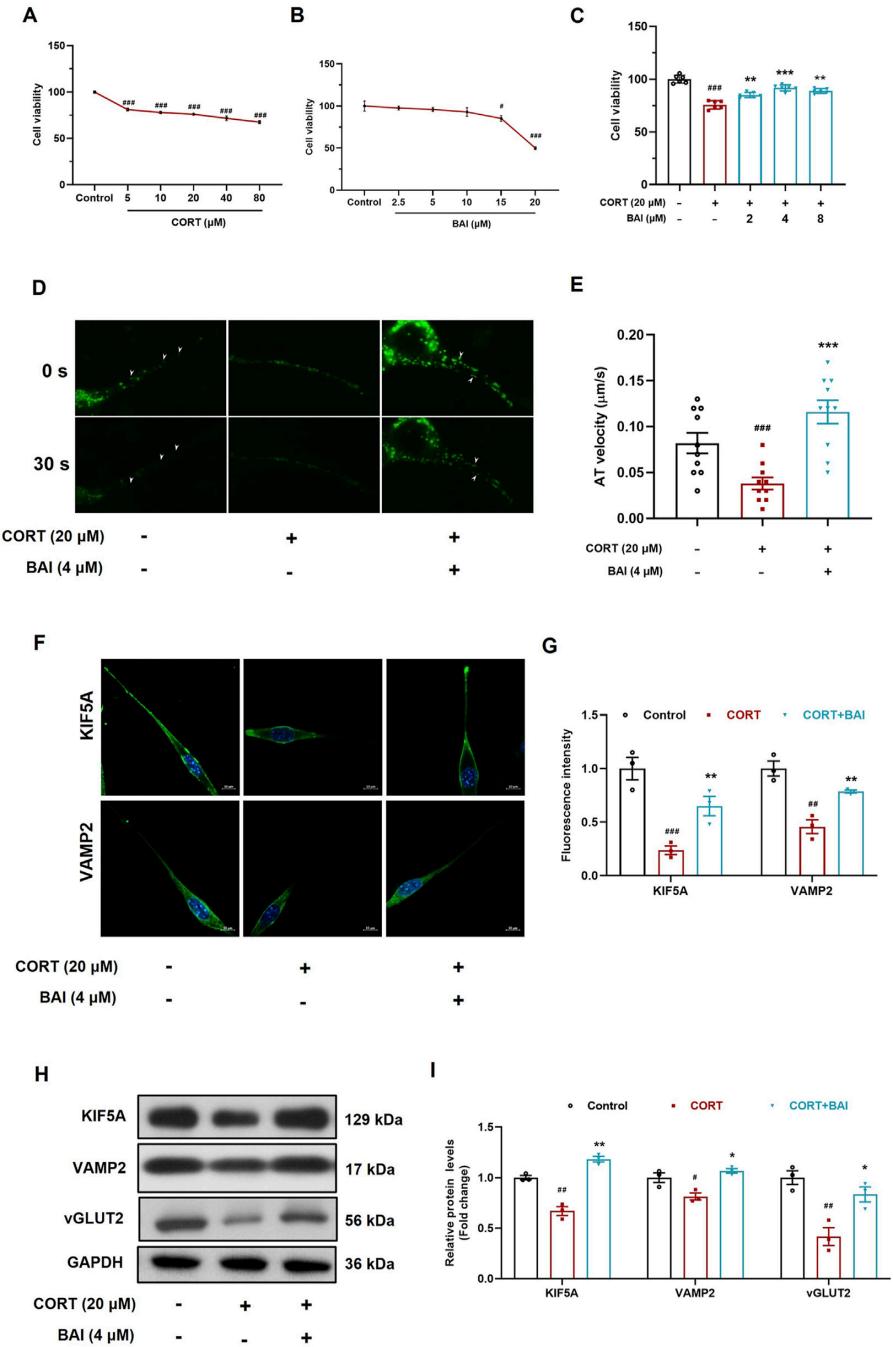
Baicalin enhances axoplasmic transport activity in HT22 cells. **(A)** Cell viability after treatment with CORT (5–80 μM) for 24 h. **(B)** Cell viability after treatment with baicalin (2.5–20 μM) for 24 h. **(C)** Cell viability after co-treated with baicalin (2, 4, 8 μM) and CORT (20 μM) for 24 h. **(D)** FM1-43 labeling of vesicle movement (white arrows indicate moving vesicles, 30 s video was accelerated to 5 s as shown in the supplementary video). **(E)** Quantification of vesicle anterograde transport velocity (10 vesicles were randomly selected and analyzed). **(F)** Immunofluorescence analysis of KIF5A and VAMP2 proteins. **(G)** Quantification of KIF5A and VAMP2 fluorescence intensity. **(H)** Immunoblot analysis of KIF5A, VAMP2, and vGLUT2. **(I)** Quantification of KIF5A, VAMP2, and vGLUT2 proteins (n = 3). Data are presented as means ± SEM and analyzed by one-way ANOVA with Tukey’s *post hoc* test. ^#^ *p* < 0.05, ^##^ *p* < 0.01, and ^###^ *p* < 0.001 compared with control group; *p < 0.05, **p < 0.01, and ***p < 0.001 compared with CORT group.

To further validate the effect of baicalin on vesicular transport, HT22 mouse hippocampal neuronal cells were cultured *in vitro*. CORT treatment significantly reduced cell viability at concentrations exceeding 5 μM (Figure 6A), whereas baicalin reduced cell viability only at concentrations exceeding 15 μM (Figure 6B). Further analysis revealed that baicalin (2–8 μM) significantly alleviated cellular injury induced by 20 μM CORT, with the optimal protective effect observed at 4 μM (Figure 6C). Consequently, 20 μM of CORT and 4 μM of baicalin were selected for subsequent experiments.

To dynamically observe the effect of baicalin on vesicular transport, we employed FM1-43 labelling to track vesicle movement. The results demonstrated that CORT treatment significantly inhibited vesicle movement, while baicalin treatment notably improved vesicle transport (Figures 6D, E; Supplementary Videos). Additionally, CORT reduced the distribution of VAMP2 and KIF5A proteins along axons, an effect that was reversed by baicalin treatment (Figures 6F, G). Furthermore, baicalin significantly increased the protein expression of both KIF5A, VAMP2 and vGLUT2 (Figures 6H, I), indicating that baicalin promotes vesicular transport and enhances the accumulation of vesicles at synaptic sites.

## 4 Discussion

Depression is primarily characterized by disrupted neuronal signaling and impaired synaptic transmission, where axoplasmic transport plays a critical role in maintaining neuronal communication and synaptic function. While previous studies have extensively demonstrated the antidepressant mechanisms of baicalin from multiple perspectives, including anti-inflammatory activity (Guo et al., 2019; Yi et al., 2024), mitochondrial protection (Jin et al., 2023), antioxidant stress responses (Zhong et al., 2019), and BDNF upregulation (Jia et al., 2021), its influence on axoplasmic transport has not been investigated. In this study, we further confirmed the antidepressant-like effects of baicalin in the CUMS model, evidenced by significant improvements in depression-like behaviors, alterations in the morphology of Nissl bodies in the hippocampus, and increased dendritic spine density—findings consistent with previous reports. The main finding of this study is the first demonstration that baicalin upregulates KIF5A expression and enhances axoplasmic transport in glutamatergic neurons, which may contribute to its antidepressant effects.

The hippocampus, particularly the CA3 region, is closely associated with emotional regulation but is especially vulnerable to stress (Kim et al., 2015). Extensive evidence implicates abnormal hippocampal synaptic structure and function in the pathophysiology of depression (Campbell and Macqueen, 2004; Videbech and Ravnkilde, 2004; Xu et al., 2020). In our study, we used TEM to examine the ultrastructure of synapses in the hippocampal CA3 region and found that baicalin significantly increased the number of presynaptic vesicles. To validate this observation, we isolated synaptosomes and confirmed that baicalin enhanced the enrichment of VAMP2, a specific synaptic vesicle marker, in the presynaptic terminals. This result further supports the notion that baicalin enhances vesicular accumulation at presynaptic sites. However, the specific type of neurons involved remains unidentified, and the underlying mechanism by which baicalin induces vesicular enrichment, as well as whether this process contributes to its antidepressant effects are also unclear.

To address these questions, we conducted transcriptomic analysis and found that DEGs were significantly enriched in glutamatergic synapses, suggesting that glutamatergic neurons may be involved. Notably, baicalin significantly increased the expression of Slc17a6, a gene encoding vGLUT2, a key marker of glutamatergic neurons. vGLUT2 plays a crucial role in the vesicular packaging of glutamate, ensuring the proper storage and release of glutamate at synaptic terminals (Eriksen et al., 2020; Zhao et al., 2023). Increased vGLUT2 expression has been associated with enhanced synaptic vesicle accumulation at presynaptic sites, which may be crucial for effective neurotransmission in glutamatergic neurons. Based on this, we hypothesize that the neurons involved may indeed be glutamatergic. We further validated this hypothesis using immunoelectron microscopy, which showed that baicalin increased the accumulation of vGLUT2-labeled vesicles at the presynaptic membrane. Taken together, these results suggest that baicalin promotes presynaptic vesicle accumulation in glutamatergic neurons by modulating glutamatergic synaptic function.

The accumulation of vesicles at the presynaptic membrane could result either from inhibited vesicle release or from enhanced axoplasmic transport. To determine the underlying mechanism, we examined several postsynaptic proteins, including PSD95, and the glutamate receptors GluA1 and GluN2B, which are essential for excitatory synaptic transmission. Their expression levels reflect the activation status of the postsynaptic membrane (Akgül and McBain, 2020; Zhang et al., 2013). We found that baicalin significantly increased the expression of these proteins, indicating that baicalin did not impair postsynaptic responsiveness. These results suggest that baicalin does not inhibit vesicle release, making it likely that the increased vesicles at presynaptic sites result from enhanced vesicle transport. Based on these findings, we further hypothesize that baicalin improves axoplasmic transport, facilitating the anterograde movement of vesicles from the soma to the synaptic terminals.

Axoplasmic transport, which is crucial for the delivery of cellular components along axons, plays a pivotal role in maintaining synaptic function. The kinesin family of motor proteins, including KIF5A, KIF5B, and KIF5C, mediates anterograde transport, while dynein is responsible for retrograde transport (Shah et al., 2022; Shi et al., 2010). To investigate whether baicalin promotes axonal transport, we evaluated the mRNA and protein expression of key motor proteins and found that baicalin significantly upregulated KIF5A expression. Previous studies have demonstrated that chronic stress reduces KIF5A levels, while fluoxetine enhances axonal transport by upregulating KIF5A (Zavvari and Nahavandi, 2020). The improvement in axonal transport facilitates the delivery of neurotrophic factors and neurotransmitters, such as BDNF, which play critical roles in mood regulation and synaptic plasticity (Hong et al., 2024).

To further validate the effect of baicalin on axoplasmic transport, we cultured HT22 cells *in vitro* and used FM1-43, a fluorescent dye that labels vesicle membranes, to dynamically track vesicle movement (Rampérez et al., 2019). Our results showed that CORT significantly inhibited vesicle movement, whereas baicalin treatment markedly enhanced vesicular transport. Additionally, baicalin increased the protein levels and axonal distribution of KIF5A, VAMP2, and vGLUT2, further supporting the notion that baicalin enhances KIF5A-mediated axoplasmic transport, thereby facilitating vesicle movement.

Taken together, our findings suggest that baicalin exerts antidepressant-like effects by enhancing KIF5A-mediated axoplasmic transport in glutamatergic neurons, offering new insights into the molecular mechanisms underlying its antidepressant effect. However, several limitations remain. First, we did not include the fluoxetine group in our mechanistic investigations, as previous studies have demonstrated that fluoxetine has no significant effect on axonal transport or VAMP2 expression in the hippocampus and synaptosomes (Bonanno et al., 2005; Jung et al., 2014). Therefore, we used fluoxetine solely as a positive control to evaluate the antidepressant efficacy of baicalin, rather than to compare the similarities or differences in their antidepressant mechanisms. Second, we did not assess the effects of baicalin on electrophysiological activities, including long-term potentiation (LTP) and long-term depression (LTD.), to further validate its effect on synaptic activity. Finally, we did not investigate whether baicalin has a similar effect on promoting axonal transport in normal neurons without CORT treatment. These aspects will be the focus of our future research.

## 5 Conclusion

In conclusion, our findings demonstrate that baicalin can improve anterograde axoplasmic transport function in the hippocampus, particularly in glutamatergic neurons, likely through the upregulation of KIF5A. This enhances synaptic vesicle trafficking, improving synaptic function and alleviating depression-like behaviours. These results provide valuable insights into the molecular mechanisms underlying antidepressant effects of baicalin and further support KIF5A as a potential therapeutic target for the treatment of depression.

## Data Availability

The datasets presented in this study can be found in online repositories. The names of the repository/repositories and accession number(s) can be found in the article/Supplementary Material.
