# Supplementary material for: A novel antidepressant mechanism of baicalin: enhancing KIF5A-mediated axoplasmic transport and vesicular trafficking in glutamatergic neurons
Source: Front Pharmacol. 2025 Apr 1;16:1577676. doi: 10.3389/fphar.2025.1577676 (PMC12023265; doi:10.3389/fphar.2025.1577676)
Supplement: Supplementary file 4 [file Image1.pdf]

Supplementary Material

1 Supplementary Figures

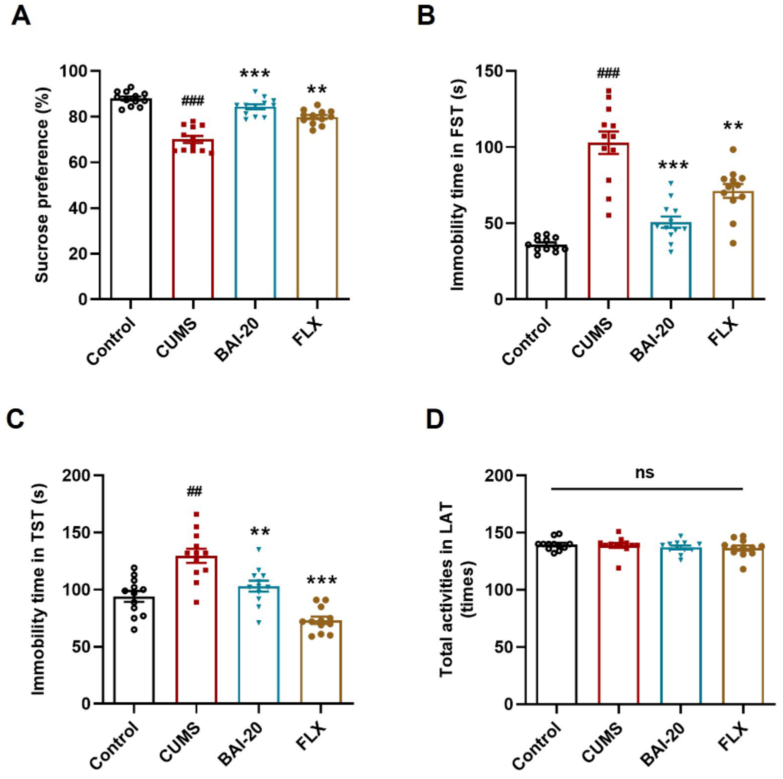

Supplementary Figure 1. Effects of baicalin on depression-like behavior in the second batch of mice induced by CUMS. (A) Sucrose preference. (B) Immobility time in the FST. (C) Immobility time in the TST. (D) Total number of locomotor activity in the LAT. Data are presented as means ± SEM and analyzed by one-way ANOVA with Tukey's *post hoc* test. n = 12. $^{\#\#}p < 0.01$ and $^{\#\#\#}p < 0.001$ compared with control group; $^{**}p < 0.01$ and $^{***}p < 0.001$ compared with CUMS group. ns: no statistical significance.

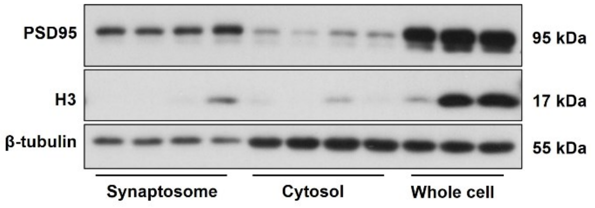

Supplementary Figure 2. Identification of hippocampal synaptosome extraction. PSD95 is enriched in synaptosomes, while H3 is exclusively detected in whole-cell lysates.

2 Supplementary Videos

HT22 cells were seeded at a density of 4×10^4 cells per dish in 20 mm dishes for 24 h. Subsequently, the cells were co-treated with baicalin (4 μM) and corticosterone (CORT, 20 μM) for an additional 24 h. Vesicle movement was visualized using FM1-43 labeling, and a 30-second video recording of the process was accelerated to 5 seconds for observation.
